# Toxicology, Nanotoxicology and Occupational Diseases Related to Chemical Exposure

**DOI:** 10.3390/ijms23169201

**Published:** 2022-08-16

**Authors:** Marina P. Sutunkova

**Affiliations:** Yekaterinburg Medical Research Center for Prophylaxis and Health Protection in Industrial Workers, 620014 Yekaterinburg, Russia; marinasutunkova@yandex.ru

The Special Issue “Toxicology, Nanotoxicology and Occupational Diseases” of the International Journal of Molecular Sciences includes six articles presenting the results of recent experimental studies in the fields of toxicology, nanotoxicology, and occupational health.

The purpose of the Special Issue is to highlight the use of various approaches for assessing the particle size-dependent toxicity of chemicals.

Challenges of workplace and ambient air pollution with emissions from mining, metallurgical, chemical and other industries remain highly relevant today. Factors of the working environment can lead to occupational and environmental diseases and adverse health effects. With regard to the chemical factor, it is important to note the absence of high-performance filtration systems ensuring 100% air purification. Industrial workers face the greatest risk levels, as they are exposed to numerous by-products, the most hazardous of which are ultrafine particles (<100 nm in size) that enter the environment and jeopardize the general population [1]. Moreover, exposure to nanoparticles may occur due to the extensive use of nanomaterials in engineering, different sectors of national economy, and medicine.

It is critical to understand the mechanisms leading to the harmful impact of these adverse factors in order to develop appropriate techniques of predictive diagnostics, detect premorbid conditions, develop and implement preventive and rehabilitative measures.

A global trend of modern research in toxicology and nanotoxicology is the use of simple objects for establishing the toxic effects of chemicals. Mirata et al. [2] conduct a comparative in vitro study of three carcinogenic mineral fibers (crocidolite, chrysotile and erionite) in the three main macrophage phenotypes: M0 (non-activated), M1 (pro-inflammatory) and M2 (alternatively activated). In their article, the authors demonstrate that these three mineral fibers act via different toxicity mechanisms. Crocidolite exerts its toxic effects mostly due to its biodurability, reactive oxygen species (ROS) and cytokine production, and DNA damage. Biodegradable chrysotile displays toxic effects related to the release of toxic metals and the production of ROS and cytokines. Other mechanisms are involved in explaining the toxicity of biopersistent fibrous erionite, which induces lower ROS and toxic metal release but exhibits a cation-exchange capacity able to alter the intracellular homeostasis of important cations. Concerning the differences among the three macrophage phenotypes, similar behavior in the production of pro-inflammatory mediators is observed. The M2 phenotype, although known as a cell type recruited to mitigate the inflammatory state in the case of asbestos fibers and erionite, serves to support the process by supplying pro-inflammatory mediators. This work is part of a long-term Italian research project of national interest (PRIN) launched in 2017 aiming to establish biochemical mechanisms leading to adverse in vivo effects related to mineral fiber exposure. Understanding the mechanisms of toxicity and carcinogenicity of inhaled mineral fibers represents a fundamental step towards a quantitative classification of toxicity/carcinogenicity of mineral fibers for preventive medicine and the development of effective treatment strategies for workers at risk and the general population [2].

At the present stage of development of molecular biology, it is feasible to study transcriptome changes following exposure to different chemicals. Transcriptomics techniques based on next-generation sequencing (NGS) are highly efficient and sensitive and allow a simultaneous analysis of a huge amount of gene expression [3,4]. To reveal the molecular mechanisms underlying complex biological processes, transcriptomic approaches can provide crucial information [5,6,7,8,9]. In their article, Kim et al. [10] describe an integrative analysis based on transcriptomic profiling aiming to investigate the impact of toluene diisocyanate (TDI) exposure on global gene expression. Their transcriptomic analysis of human bronchial epithelial cells (BEAS-2B) after TDI treatment shows that dual-specificity phosphatase 6 (DUSP6) is one of the genes significantly changed by TDI exposure. TDI exposure also increases the mRNA level of p53 along with its protein and activity which trans-activates DUSP6 but attenuates extracellular signal-regulated kinase 1/2 activity through transient receptor potential ankyrin 1 receptor activation. TDI exposure has a cytotoxic effect but attenuates the apoptotic response, which suggests that it promotes the survival of cancer cells.

Occupational exposure to industrial chemicals is usually associated with muscular work, especially in mining and metallurgy. The combined effect of such factors on the development of pathological processes has been studied, but data are scarce. In case of inhalation exposure, the absorbed dose is bound to increase owing to more intensive lung ventilation; however, one may expect that the extent of the response to unit dose may vary in either direction under the effect of various mechanisms related to muscle work. It has been demonstrated, for instance, that in rats with developing experimental silicosis, this process may be both enhanced and attenuated when the animals are forced to run every day, depending on the running speed [11,12]. The Special Issue presents yet another work designed to establish the combined effect of physical activity and lead exposure on the cardiovascular system of rats [13]. The authors find that this repeated short-term running exercise somewhat attenuates the toxic and cardiotoxic effects of lead. The physical activity of animals levels the lead-induced molecular disturbances to the composition and properties of the contractile proteins of the heart. This means that low- and moderate-intensity running can be recommended as a measure of lead poisoning prevention.

The current progress in the global nanotechnology industry is aimed at creating new and efficient diagnostics and unique therapeutic nanosized agents. This is possible thanks to biospecific properties of nanoparticles “attached” to polymers intended for specific delivery and the binding of nanoparticles to biological targets. Outcomes related to physical, chemical and biological effects will allow us to solve most diagnostic and therapeutic tasks [14,15,16]. Novikov et al. [17] carry out a comparative assessment of the biological effects of silver nanoparticles encapsulated in natural and synthetic stabilizing polymer matrices and reveal the features of their effects. Pathological abnormalities expressed in the structure of the temporoparietal zone of the sensorimotor cortex of the rat brain, increasing over time, are found in the nanobiocomposites of silver nanoparticles and the natural polysaccharide arabinogalactan. At the same time, the introduction in a similar mode and dose of silver nanoparticles encapsulated in a synthetic matrix of poly-1-vinyl-1,2,4-triazole does not lead to any noticeable changes in the studied parameters early on or in the long run. Features of the biological effects of silver nanoparticles encapsulated on various matrices can be used in medical research to reduce the adverse effects of silver nanoparticles.

Nowadays, few studies have investigated combined effects of binary and complex mixtures of nanoparticles (NPs) on organisms. Previous studies of combined toxicity have used bacteria [18], microalgae [19,20], crustaceans, and fish [21] for the risk assessment of binary mixtures of NPs. In general, the impact of suspended particles or dissolved metal ions and the interaction between mixtures of NPs and aquatic organisms of different trophic levels remain uncertain, and this issue requires further study.

In their combined ecotoxicity study, Pikula et al. [22] assess the toxicity of binary mixtures of nanoparticles and reveal that the toxicity of mesoporous silicon dioxide with metal inclusions (SMB24) is reduced in the presence of CdS and ZnS by the probable scavenging of additional Zn^2+^ ions and ZnO inclusions from SMB24. The tested TiO_2_ and mesoporous silicon dioxide sample without any inclusions of SMB3 demonstrates synergistic toxic action in all the combinations with the other NPs except SMB24. The supposed reason for the synergistic toxic action of TiO_2_ and SMB3 combined with the other NPs is a “Trojan horse effect”, which increases the bioavailability of the other metal-containing NPs for aquatic organisms. Further studies on environmental toxicology should include more species from different trophic levels, more types of nanoparticles and their combinations, broader biomarker and target characteristic boards, omics approaches, and chronic toxicity bioassays with low doses of toxicants.

Another study on the evaluation of the combined toxicity of nanoparticles is presented by Minigalieva et al. [23] in their article describing the application of the classification of combined three-factor toxicity based on a two-stage assessment approach considering: (1) the type of combined effect of two agents; (2) the type of combined effect of the same two agents against the effect of the third one. Such an approach enables the authors to identify changes in the type of binary toxicity following the addition of a third agent and to establish that the outcomes of exposure to a ternary mixture of NPs can be more or less noxious (Classes A and B, respectively) or remain unchanged (Class C) following the addition of a third component. When assessing the cumulative health risk from exposure to a ternary mixture of toxicants based on the generally accepted approach of single-factor risk summation, it is essential to take into consideration that the result of such an assessment may be either underestimated (if the toxicity effects fall into Class A) or somewhat overestimated (if they fall within Class B). The first option is more important from the perspective of the precautionary principle, while the second should be considered only as an additional margin of safety. The developed classification can be used to assess human health risks from combined exposure to three types of nanoparticles and nanomaterials.

This Special Issue contributes to the dissemination of the main findings of recent research in the field of toxicology, environmental toxicology, nanotoxicology, and health risk assessment.
